# Assessment of the Accuracy of Cardiopulmonary Resuscitation Videos in English on YouTube according to the 2015 AHA Resuscitation Guidelines

**DOI:** 10.1155/2019/1272897

**Published:** 2019-05-02

**Authors:** Burak Katipoğlu, İlker Akbaş, Abdullah Osman Koçak, Muhammet Furkan Erbay, Engin İhsan Turan, Kamber Kasali

**Affiliations:** ^1^Department of Emergency Medicine, Ufuk University Faculty of Medicine, Ankara, Turkey; ^2^Department of Emergency Medicine, Ataturk University Faculty of Medicine, Erzurum, Turkey; ^3^Department of Biostatistics, Ataturk University Faculty of Medicine, Erzurum, Turkey

## Abstract

Over the last decade, YouTube has become one of the largest online resources for medical information. However, uploaded videos are published without any peer review or quality control, so incorrect and incomplete information can be easily disseminated via the virtual platform and can be perceived as correct. The YouTube website was searched for videos in English uploaded between 15 October 2015 and 21 October 2016 using the following keywords: “CPR,” “cardiopulmonary resuscitation,” and “basic life support.” This study had a cross-sectional analytical design. In the first evaluation, the accuracy of the videos was checked according to the information contained in the basic cardiac life support algorithm. In the second evaluation, we assessed whether advanced-level, innovative medical information was included in these videos; when included, the accuracy of such information was checked. Of 774 videos evaluated, 92 videos were included in the study after application of the exclusion criteria. The videos were scored on a scale ranging from 0 to 20 points. The mean total score, based on all criteria, was 4.79 ± 2.88. The highest mean total score was achieved by videos uploaded by official medical organizations (6.43 ± 3.57), followed by those uploaded by health professionals and organizations (4.25 ± 2.49), and those uploaded by unidentified sources. YouTube videos are insufficient in providing information about the basic life support algorithm and advanced-level information according to the 2015 AHA resuscitation guidelines for health professionals. The educational material published by health institutions that are constantly working in the area is a more reliable source of information on subjects that directly affect human life, such as cardiopulmonary resuscitation.

## 1. Introduction

Web-based platforms that facilitate the sharing of user-generated content and information, such as social networking sites, media sharing sites, blogs, and microblogs, have changed the methods of information sharing worldwide [1]. This radical change has naturally led to several innovations for accessing information, distributing knowledge, and conducting teaching activities. The World Wide Web has become a popular medium for learning and teaching. Over the last decade, the Internet has become a major source of medical information, and different methods are being developed for medical education utilizing the Internet [2]. The increasing integration of the Internet and social media into medical education suggests that online medical training will form the basis of future medical education [3]. In addition to patients and laypeople, increasing numbers of medical students and health professionals use the Internet and social media to obtain information about their areas of specialization [4]. Used for video sharing, YouTube is not only the most popular social networking site, but also the largest video search engine on the Internet [4]. The website, established in 2005, provides users with the ability to upload, share, view, and download video clips via a simple, integrated platform [5]. Approximately 300 million hours of videos are uploaded to this site and accessed by approximately 1.3 billion people worldwide. Approximately 5 billion videos are watched every day, and a total of 3 billion hours of videos are displayed per month [6]. Although this popular website is based in the USA, 70% of its users reside outside of the USA; thus, the platform allows people to interact with individuals around the world [7]. The videos uploaded on this site include those providing information, entertainment, news, advertisements, and personal content [8]. In addition, the site provides visual content intended for educational purposes. The educational video content has been increasing steadily with the rise in the number of views [7]. Early recognition of sudden cardiac arrest and early cardiopulmonary resuscitation (CPR) are known to improve patient survival [9, 10]. Self-training in CPR through the viewing of online videos is used widely in the USA, especially for bystander CPR training [11].

Therefore, YouTube is a valuable source of information for cardiac basic life support (BLS) and CPR for healthcare professionals, as well as non-healthcare professionals and ordinary rescuers. Due to the heterogeneous nature of YouTube, rescuers can provide BLS and CPR information from helplines while providing current cardiac life support information to health professionals. YouTube videos can be used as a cost-effective and easily accessible medical training method for cardiac life support and CPR, providing good-quality, accurate, and updated content. Several studies have examined the reliability of BLS and CPR videos uploaded to YouTube [11]. These studies assessed the suitability of the visual materials in accordance with the American Heart Association (AHA) guidelines published in or before 2010. To our knowledge, no study has examined the compliance of BLS and CPR videos in English uploaded to YouTube with the updated AHA guidelines published in 2015. In addition, previously published studies have generally focused on knowledge regarding the BLS algorithm. However, the ability to include more advanced-level information in these videos has not been evaluated. This study was performed to examine the accuracy of BLS and CPR videos on YouTube according to the 2015 AHA Resuscitation Guidelines.

## 2. Materials and Methods

This study had a cross-sectional analytical design. The YouTube (YouTube^©^, https://www.youtube.com; YouTube, LLC, San Bruno, CA, USA) website was searched for English-language videos uploaded between 15 October 2015 and 21 October 2016 using the following keywords: “CPR,” “cardiopulmonary resuscitation,” and “basic life support.” The date 15 October 2015 was chosen because it is the date of publication of the 2015 AHA Guidelines for CPR and Emergency Cardiovascular Care.

Videos meeting at least one of the following exclusion criteria were excluded: nonmedical content (advertisements, news, or interviews); publication in a language other than English; pediatric CPR footage; live-action footage (real-life videos) devoid of educational content; comedy or entertaining content, except for educational purposes; duplicate footage; content promoting CPR devices; and animal CPR footage.

Information regarding the sources of the videos included in the survey, their durations, number of views during the study period, and whether human subjects were used as models was recorded. The sources of the videos, i.e., the uploaders, were divided into the following groups: official medical organizations (e.g., AHA, European Society of Cardiology, International Liaison Committee on Resuscitation (ILCOR), the European Resuscitation Council (ERC)), healthcare professionals and organizations (e.g., doctors, nurses, paramedical personnel, medical faculties, and hospitals), and unidentified (uploaders not belonging to the first two source groups and/or using unknown nicknames).

Two physicians specializing in emergency medicine watched all videos included in the study. A third physician's opinion was sought in cases of disagreement between the two emergency physicians. Two aspects of the videos were evaluated. In the first evaluation, the validity of the videos was assessed according to selected information considered to be important for the BLS algorithm (Table 1). In the second evaluation, the ability of the videos to transmit advanced-level medical information was assessed. Information pertaining to important innovations outlined in the 2015 AHA guidelines was selected for this assessment (Table 2). If this information was mentioned in a video, its accuracy was checked. If correct information was provided in accordance with the criteria, a score of 1 point was given. If the information was not provided in the video or was inaccurate, a score of 0 was given.

### 2.1. Statistical Analysis

Statistical analyses were conducted using the IBM SPSS 22 statistical software (IBM, Armonk, NY, USA). Data are presented as means, standard deviations, medians, minimums, maximums, percentages, and frequencies. The normal distribution of continuous variables was confirmed using the Shapiro–Wilk test. Analysis of variance was used to compare normally distributed continuous data from more than two groups. The Kruskal–Wallis test was used for non-normally distributed data. Thereafter, post hoc tests were performed. In all analyses,* p*<0.05 was taken to indicate statistical significance.

## 3. Results

In total, 774 English-language videos identified using the search terms “CPR,” “cardiopulmonary resuscitation,” and “basic life support” from among all videos uploaded to YouTube between 15 October 2015 and 21 October 2016 were evaluated. Of these videos, 682 were excluded based on the exclusion criteria. The number of excluded videos and the reasons for exclusion are shown in Table 3. Of the excluded videos, 441 (64.6%) contained the key term “basic life support,” 75 (11%) had the key term “cardiopulmonary resuscitation,” and 166 (24.34%) had the key term “CPR.”

The analysis included 92 videos that fulfilled the inclusion criteria. Of the included videos, 48 (52.2%) contained the key term “CPR,” 26 (28.3%) had the key term “cardiopulmonary resuscitation,” and 18 (19.6%) had the key term “basic life support.” The majority (*n*=41, 44.6%) of the videos were uploaded by unidentified sources, followed by those uploaded by healthcare professionals or organizations (*n*=28, 30.4%), and those uploaded by official medical organizations (*n*=23, 25%).

The videos ranged in duration from 26 to 5559 s (mean, 450 ± 848 s; median, 250 s). Videos longer than 6–10 min had the highest average number of views (mean, 13,495 ± 33,275; median, 275; minimum, 4; maximum, 14,496), followed by those with durations ≥10 min (median, 260; minimum, 5; maximum, 33,987) and those with durations of 1–3 min (median, 259; minimum, 4; maximum, 70,611). Video duration was not related significantly to the number of views.

Among the videos included in this study, 18.5% (*n*=17) used human subjects as models or for the demonstration of medical procedures, 39.1% (*n*=36) used mannequins, and 4.3% (*n*=4) used human subjects and mannequins. Thirty-five (38%) of the videos featured neither human subjects nor mannequins. The type of educational resource used was not related significantly to the average number of views.

No video included in the study scored 20 points.  Table 4 shows the number of videos that included information of each type. The average total scores for the videos, calculated according to the average scores shown in Tables 1 and 2, are shown in Table 5. The highest average total score was achieved by videos uploaded by official medical organizations (6.43 ± 3.57), followed by videos uploaded by healthcare professionals and organizations (4.25 ± 2.49) and those uploaded by unidentified sources (4.24 ± 2.39). The scores of the videos uploaded by official medical organizations were significantly higher than those of videos uploaded by unidentified sources (*p*=0.03).

The average number of views, average video duration, and scores are shown according to video source in Table 6. Videos uploaded by official medical institutions had significantly more views than did those uploaded by individuals without medical credentials (*p*=0.002).

The relationship between the average number of views and scores was examined. According to the criteria outlined in Table 1, videos with scores of 1–2 points were watched 9001 ± 21,558 times (median, 199; minimum, 6; maximum, 92,575), those with scores of 3–4 points were watched 14,960 ± 32,529 times (median, 962; minimum, 4; maximum, 149,996), those with scores of 5–6 points were watched 819 ± 1552 times (median, 134; minimum, 2; maximum, 6859), those with scores of 7–8 points were watched 3888 ± 9151 times (median, 257; minimum, 24; maximum, 30,881), and those with scores of 9–10 points were watched 10,345 ± 13,268 times (median, 3396; minimum, 368; maximum, 33,987). The number of views was not associated significantly with the score.

## 4. Discussion

Anyone registered on the YouTube video-sharing site can upload a video. The uploaded videos are published without peer review or quality control, as long as they are not offensive and do not contain illegal material [2]. Despite the advantages associated with the ease of information broadcasting, the dissemination of information through the Internet in general and through YouTube in particular is accompanied by significant legal, ethical, personal, and professional hazards due to the uncontrolled and possibly inaccurate information that is published [3, 12]. Due to the absence of a quality control system for this information channel, incorrect and incomplete information can easily spread via this virtual platform and may be perceived as accurate. The results of the present study suggest that YouTube videos are inadequate in providing basic information regarding the BLS algorithm and advanced-level medical information consistent with the 2015 AHA CPR guidelines to healthcare professionals.

Despite the huge volume of information available on the Internet, the unstructured format often makes it difficult to obtain required information. About 88% of the videos assessed in this study were excluded because they did not meet the inclusion criteria. Similarly, several previous studies examining the reliability of YouTube videos reported exclusion of 80%–94% of videos [4, 9, 11, 13]. As in these previous studies, we found that the high exclusion rate made it difficult to find the required content on YouTube.

The 774 videos assessed in this study were uploaded to the site within a period of 371 days. Murugiah et al. [13] reported that the average number of videos uploaded per day was 1.65, and Yaylaci et al. [11] reported a value of 1.82 per day. In the present study, this number was 2.08 per day. Researchers evaluating the compliance of YouTube videos with the resuscitation guidelines have concluded that the increasing number of average daily video uploads over the years indicates that these issues have gained in popularity.

Since the publication of the first AHA CPR guidelines in 1969, the society has published revisions every 5–6 years, ensuring that information about resuscitation remains updated. Whereas the formulation of the 2010 guidelines involved extensive revision of the previous version, the 2015 guidelines included only minor revisions, focusing mainly on controversial issues and their solutions [14]. These guidelines, formulated by medical associations under the guidance of the International Liaison Committee on Resuscitation, mainly the AHA, are followed carefully in medical practice and are widely anticipated. The present study showed that videos on resuscitation that were uploaded by national and international associations were viewed more often than those uploaded by unidentified sources. Similar results were reported by Sasmaz et al. [4], based on their research on trauma management videos, and by Yaylaci et al. [11], based on two trials examining CPR videos. The greater number of views for videos uploaded by official medical organizations compared with those uploaded by other sources suggests a greater level of trust in these associations. Therefore, official associations should assume greater responsibility for disseminating not only CPR-related, but also other types of health-related, information using the Internet.

No video included in this study achieved a perfect score of 20 points. To assess compatibility with published information, we selected the 2015 BLS algorithm, and the median score was 4/10. Yaylaci et al. [11] investigated compliance of the information in YouTube videos with the 2010 CPR guidelines in a similar study and reported a median score of 5/7; they emphasized the potential good educational outcomes of the use of these videos. The low scores in the present study indicated that the videos assessed were not consistent with the 2015 CPR guidelines. In contrast to the findings of Yaylaci et al. [11], our results suggest that the dissemination of information related to BLS and CPR via YouTube videos can lead viewers to acquire inadequate, incomplete, and inaccurate knowledge.

This study was performed to assess the suitability of YouTube videos for the dissemination of theoretical information for health professionals. The average scores for the videos taken from Table 2 were 0.37 ± 1.09 (median, 0; minimum, 0; maximum, 8). The videos had a maximum score of 7 points in this section, and only one video fulfilled four criteria. These results indicate that, despite the provision of insufficient basic information, the visual material is relatively useful. These findings also indicate that the videos contained incomplete and incorrect advanced-level medical information for health professionals.

The average total score of the videos uploaded by official medical organizations (6.43 ± 3.57) was significantly higher than that of videos uploaded by unidentified sources (4.24 ± 2.39), consistent with previous reports [4, 9, 11]. With regard to information related to topics that directly affect human life, such as CPR, materials published by institutions that are constantly working in this field are more reliable. These types of video that are relevant to human life should be moderated and authenticated by academic supervisors before being published on YouTube.

The scores obtained in the first evaluation were not related significantly to the number of views of the videos included in this study. Although we hypothesized that high-scoring videos would be viewed more often, previous studies have yielded findings similar to ours [4, 11]. People cannot gain access to the visual content without viewing the video. However, scoring of the videos prior to publication on the site would allow the audience to decide whether to view the videos based on their scores, which could change the association between the score and the number of viewers.

The average duration of the 50 most-watched videos on YouTube is 2 min 54 s [15]. Thus, the attention span of the YouTube audience is long (about 3 min), and interaction decreases after 5 min [16]. The median duration of the videos in our study was 250 s, which was longer than that of the most popular videos. Yaylaci et al. [11] reported a median video duration of 160 s. The median score of the videos in their study was higher than that of the videos in our study [11]. Shorter videos have greater numbers of views on YouTube. Therefore, medical organizations will be able to reach wider audiences by publishing shorter videos.

The main limitation of this study is that inclusion criteria and scoring criteria used in our study were subjective. We evaluated only videos in English for consistency with the 2015 AHA Resuscitation Guidelines and did not take into account other resuscitation guidelines (e.g., ERC Guidelines for Resuscitation 2015, Australian and New Zealand Committee on Resuscitation guideline 2016). These guidelines and AHA guideline are local adaptations of the evidence previously published in October 2015 by the ILCOR [17]. There are no significant differences in other guidelines according to the 2015 AHA guideline, except in the use of IM/IN naloxone in opioid overdose. The parameters used in evaluating the suitability of the videos are not in contradiction with other guidelines.

## 5. Conclusions

The results presented here showed that CPR- and BLS-related videos in English published on YouTube during the study period were not in compliance with the 2015 AHA guidelines in terms of providing basic information to the general population. In addition, health professionals cannot obtain advanced-level medical information via these videos. The observation that videos uploaded by official medical organizations had greater numbers of views suggests that people have greater trust and confidence in these institutions. A mechanism by which such educational videos are audited before they are uploaded to YouTube would ensure the dissemination of accurate, complete, and useful health-related information and would increase the number of views of such videos.

## Figures and Tables

**Table 1:** Criteria selected from the basic life support algorithm used for video evaluation.

Information selected from the basic life support algorithm
(1) Providing environmental safety (2) Control of patient non-response (3) Achieving airway clearance and evaluation of respiration (4) Activation of emergency medical system using mobile devices (5) C-A-B sequence (6) 30:2 chest compression (7) Correct localization for chest compression (8) Appropriate chest compression depth (5–6 cm) (9) Use of defibrillator (10) Chest compression should be 100–120/min

**Table 2:** Selected innovations mentioned in the 2015 American Heart Association guidelines used for video evaluation.

Innovations selected from the 2015 AHA guidelines
(1) Ventilation should be done 10 times per minute in those with advanced airway (2) Public access defibrillation program (3) IM/IN naloxone use in opioid overdose (4) Compression/fraction >60% (5) CPR guided by the emergency medical system (6) End-tidal CO_2_ value in the intubated patients <10 mmHg at the end of 20 minutes; this is one of the parameters that can be used to terminate CPR (7) The use of vasopressin is no longer recommended in the guidelines (8) Adrenaline can be used early in unshockable rhythms (9) Extracorporeal CPR recommendation (10) Delayed ventilation

**Table 3:** Number of excluded videos.

Reason for the exclusion	*n* (%)
Presence of non-medical content (advertisements, news, and interviews)	374

Language other than English	109

Pediatric cardiopulmonary resuscitation footage	43

Lack of educational content, presence of live action footage (real-life videos)	30

Comedy and entertainment content	68

Duplicated footage	43

Footage of cardiopulmonary resuscitation of animals	15

Total	682

**Table 4:** Number of videos containing information for each criterion.

Information required in the study	Number of videos containing the required information (%)
Providing environmental safety	33 (35.9%)

Control of patient's non-response	58 (63%)

Achieving airway clearance and evaluation of respiration	39 (42.4%)

Activation of emergency medical system using mobile devices	48 (52.2%)

C-A-B processing	39 (42.4%)

30:2 chest compression	55 (59.8%)

Correct localization for chest compression	52 (56.6%)

Appropriate chest compression depth (5–6 cm)	21 (22.8%)

Use of defibrillator	37 (40.2%)

Chest compression rate should be 100–120/min	26 (28.3%)

Ventilation should be done 10 times per minute in those with advanced airway	(1.1%)

Public access defibrillation program	5 (5.4%)

IM/IN naloxone use in opioid overdose	5 (5.4%)

Compression/fraction >60%	(54%)

CPR guided by the emergency medical system	8 (8.7%)

End-tidal CO_2_ value in the intubated patients <10 mmHg at the end of 20 minutes; this is one of the parameters that can be used to terminate CPR	1 (1.1%)

The use of vasopressin is no longer recommended in the guidelines	4 (4.3%)

Adrenaline can be used early in unshockable rhythms	3 (3.3%)

Extracorporeal CPR recommendation	1 (1.1%)

Delayed ventilation	1 (1.1%)

**Table 5:** Total average scores of the videos included in this study

	Mean	Standard Deviation	Median	Minimum	Maximum
Score from Table 1 (0–10)	4.435	2.491	4	0	10

Score from Table 2 (0–10)	0.370	1.097	0	0	8

Total score (0–20)	4.793	2.884	4	0	17

**Table 6:** Average number of views, average video duration, and scores according to video source.

	Uploaded by official medical organizations	Uploaded by healthcare professionals or organizations	Uploaded by unidentified sources	All videos
Average views	14145 ± 26332 median: 2228 (2–92575)	7999 ± 11882 median: 1052 (5–3374)	5040 ± 24168 median: 134 (4–149996)	8252 ± 21873 median: 275 (2–149996)

Scores as per first evaluation*∗*	5.70 ± 2.72 median: 5 (2–10)	3.89 ± 2.44 median: 4 (0–9)	4.10 ± 2.19 median: 4 (0–10)	4.43 ± 2.49 median: 4 (0–10)

Scores as per second evaluation*∗∗*	0.78 ± 1.73 median: 0 (0–8)	0.36 ± 1.06 median: 0 (0–4)	0.15 ± 0.42 median: 0 (0–2)	0.37 ± 1.09 median: 0 (0–8)

Average total score	6.43 ± 3.57 median: 6 (2–17)	4.25 ± 2.49 median: 4 (0–9)	4.24 ± 2.39 median: 4 (0–12)	4.73 ± 2.88 median: 4 (0–17)

*∗* Scores of videos according to the criteria selected from the basic cardiac life support algorithms.

*∗∗* Scores of videos based on innovations from the 2015 American Heart Association guidelines.

## Data Availability

The SPSS/Excel data are used to store the findings of this study. Data are available from Burak Katipoğlu, Department of Emergency Medicine, Ufuk University, Ankara/Turkey. Please mail us on burak44katipoglu@gmail.com for researchers who meet the criteria for access to confidential data.
